# Improved survival of thalassaemia major in the UK and relation to T2* cardiovascular magnetic resonance

**DOI:** 10.1186/1532-429X-10-42

**Published:** 2008-09-25

**Authors:** Bernadette Modell, Maren Khan, Matthew Darlison, Mark A Westwood, David Ingram, Dudley J Pennell

**Affiliations:** 1 UCL Centre for Health Informatics and Multiprofessional Education (CHIME), Holborn Union Building, Whittington Campus, Highgate Hill, London, N19 5LW, UK; 2 CMR Unit, National Heart and Lung Institute, Imperial College & Royal Brompton Hospital, London, SW3 6NP, UK

## Abstract

**Background:**

The UK Thalassaemia Register records births, deaths and selected clinical data of patients with thalassaemia who are resident in the UK. A study of survival and causes of death was undertaken which aimed to include the possible impact of T2* cardiovascular magnetic resonance (CMR).

**Methods:**

The Register was updated to the end of 2003, copies of death certificates were obtained, and causes of death in beta thalassaemia major were extracted. In addition, patients who had T2* CMR assessment of cardiac iron load and/or received the oral iron chelator deferiprone were identified from clinical records.

**Results:**

The main causes of death were anaemia (before 1980), infections, complications of bone marrow transplantation and cardiac disease due to iron overload. From 1980 to 1999 there were 12.7 deaths from all causes per 1,000 patient years. Forty per cent of patients born before 1980 had T2* cardiovascular magnetic resonance between 2000 and 2003, and 36% of these patients were prescribed deferiprone before end of 2003. In 2000–2003, the death rate from all causes fell significantly to 4.3 per 1,000 patient years (-62%, p < 0.05). This was mainly driven by the reduction in the rate of deaths from iron overload which fell from 7.9 to 2.3 deaths per 1,000 patient years (-71%, p < 0.05).

**Conclusion:**

Since 1999, there has been a marked improvement in survival in thalassaemia major in the UK, which has been mainly driven by a reduction in deaths due to cardiac iron overload. The most likely causes for this include the introduction of T2* CMR to identify myocardial siderosis and appropriate intensification of iron chelation treatment, alongside other improvements in clinical care.

## Introduction

Over the past 50 years the prevalence of thalassaemia major has increased throughout North-West Europe, reflecting immigration from endemic areas [1]. In the United Kingdom (UK) a database of affected children was created in the 1960s to assess the prevalence of the disorder among Cypriots in London [2], and to describe its natural history [3]. This was subsequently developed into the UK Thalassaemia Register, a clinical and research tool designed to promote equitable high quality care by simultaneously collecting audit data, and disseminating specialist knowledge to clinics with small numbers of patients. The Register attracted a very high level of collaboration from doctors treating patients with thalassaemia, and is considered to be over 97% complete [4].

Regular transfusion to maintain a mean haemoglobin in the normal range, which became standard treatment in the 1960s [3], gives good quality of life in the short term, but leads to death from transfusional iron overload at between 12 and 24 years of age. In the UK, the iron chelating agent deferoxamine became available in 1964 [5]. Daily intramuscular injection of 20–25 mg/kg/day stabilised body iron load at around 0.65 mg/kg body weight [6], and improved survival [7]. In 1976 it was shown that subcutaneous infusion of a higher dose using a portable syringe driver could stabilise body iron load at around 0.3 mg/kg body weight [8], and by 1980 subcutaneous infusion of an average daily dose of 40–45 mg/kg/day (usually over 8 to 12 hours on five nights per week) had become standard care in the UK. Bone marrow transplantation for thalassaemia was initiated in the UK in 1984 [9], and has been requested by most families with a compatible related donor since the mid-nineties [4]. The oral iron chelating agent deferiprone was first used in clinical practice in 1987 [10], and its use in the UK has gradually increased, particularly for patients who cannot tolerate deferoxamine treatment [11], and for patients with cardiac iron loading. A second oral iron chelator, Deferasirox, was introduced into clinical trials in the UK only after the data reported here was collected. The reliable identification of cardiac siderosis was made possible with the T2* cardiovascular magnetic resonance (CMR) technique which became available in the UK in 1999 [12], and this has been a driver for changes in, and intensification of, iron chelator therapy. Also in 1999, the UK Register information on continuing high mortality in thalassemia was circulated to all collaborating doctors. These data were published 18 months later [7]. The poor patient survival was disappointing given the general availability of deferoxamine treatment, and was considered largely due to non-compliance to the burdensome iron chelation regimen among adolescent and young adult patients. However, cardiac disease and death had also occurred among compliant patients with a low serum ferritin and low liver iron concentration, suggesting that these measures are not always a reliable guide to cardiac iron load [12-14]. The communication indicated that improved methods for administering deferoxamine and availability of the oral iron chelating agent deferiprone now allowed more tolerable patient-specific iron chelation regimens, and reinforced the existing recommendation to refer patients regularly to an expert centre for assessment and advice. In the light of these events of 1999, we analysed of the frequency and the causes of death for before and after this year.

## Methods

The UK Thalassaemia Register provides a minimum data set, drawn from the clinical records of patients with thalassaemia. The scientific method is fully described elsewhere [4]. Underpinning informatics methods are aligned with current approaches to electronic healthcare records, with a view to integration in the long-term. Ethical approval for this study was obtained.

### Numbers of deaths

At the beginning of 2003, participating doctors were requested to update the current status of their patients (alive, transferred away, new patient, deceased), and at the end of 2003 they were asked to notify any deaths that had occurred during the year. Thus at the end of 2003 information on number of deaths was complete (though information on new births was incomplete because it usually takes 1–3 years for a patient to present and be registered). The register has not been further updated since this time primarily because of funding issues, and therefore no further data after the end of 2003 is currently available.

### Causes of deaths

The Register already held a summary of the entire clinical record and a copy of the death certificate for deaths prior to 1981 [3]: causes of death identified from the notes and from the death certificate were consistent. Copies of the remaining death certificates were supplied by the Office of National Statistics. UK death certificates include three sections for cause of death (eg cardiomyopathy due to iron overload due to thalassaemia major). This was fully completed in all but one case, where the only cause of death given was "thalassaemia major". All causes were entered in the Register. When the cause was infection unrelated to bone marrow transplantation, the patient's doctor or hospital was asked for any available information on the organism responsible.

### Calculation of death rates

Data was analysed by 10-year birth cohort to the end of 1999, and for the four years 2000 to the end of 2003. Mortality rate (deaths per 1,000 patient years) was calculated for each birth cohort for each decade or part of a decade. The significance of differences in death rates was assessed by calculating 95% confidence intervals: absence of overlap is considered statistically significant at the usual 5% level.

### Calculation of life-expectancy

Patients alive at the beginning of each decade were classified by 5-year age groups (0–4, 5–9 etc). The percentage in each age group who died in the next 5 years was calculated, and cumulative mortality (survival) was presented graphically.

### CMR

T2* CMR was performed using standard techniques as first described by Anderson [12], and refined by Westwood [15] and Maceira [16]. Cardiac siderosis was defined as a myocardial T2* < 20 ms [12].

### Deployment of deferiprone and CMR

T2* cardiovascular magnetic resonance (CMR) was introduced in 1999 at Royal Brompton Hospital in London, where it was first developed [12]. T2* CMR was applied rapidly in clinical management from 2000 as the benefits became clear of direct visualisation of cardiac siderosis as a guide to the need for, and a means of assessing the response to intensified iron chelation therapy. Increased iron chelation was deployed with intensified deferoxamine or deferiprone [17], as monotherapy or in combination, by clinicians in consultation with individual patients. Increasingly the decision to treat with deferiprone was made in response to the finding of cardiac iron loading. To document the deployment of CMR and deferiprone, data was extracted on patients with thalassaemia who had had CMR by end-2003, and the iron chelation therapy they were receiving at the time.

## Results

### Survival analysis

The UK Thalassaemia Register includes all patients with a serious thalassaemia (Table 1). However, this report is limited to the 850 recorded patients with thalassaemia major (transfusion dependent before four years of age). Table 2 shows the causes of the 206 deaths in this group by decade. The commonest cause of death was iron overload (111 cases, 54% of total) with all death certificates reporting a cardiac cause. There were 19 deaths (9% of total) from infection. The organism responsible was known in only seven cases: there were two deaths from Yersinia infection, one from pneumococcal septicaemia, and four from Klebsiella meningitis. Thirty three patients (16% of total) died from anaemia, but only 3 of these deaths occurred after 1975: one 17-year-old declined further treatment, and 2 were young children whose parents took them for an extensive stay abroad. Death due to complications of bone marrow transplantation (21 patients, 10% of total) was first reported in 1984 and has now become rare. There were two deaths (1%) due to malignancy in young children (one leukaemia, one medulloblastoma). More recently, two patients (1%) with hepatitis C infection died from hepatoma aged 39 and 47. One woman (0.5%) died aged 39 from carcinoma of the breast. The 16 deaths (8%) due to "other causes" included three from liver disease, three from disturbed coagulation (two bleeding, one clotting), two from diabetes, two from renal failure, one from congenital heart disease, one from lung disease, one from cardiac tamponade caused by a misplaced Hickman line, two due to transfusion of infected blood, and one due to a road traffic accident.

**Table 1:** Patients on the UK Thalassaemia Register at the end of 2003.

**Diagnosis**	**Alive**	**Dead**	**Total**
Beta thalassaemia major	644	206	850
Beta thalassaemia intermedia	138	20	158
Hb E/beta thalassaemia	73	7	80
Alpha thalassaemia major	1	0	1

Total	856	233	1,089

Alpha thalassaemia major = homozygous alpha zero thalassaemia

**Table 2:** Changes over time in numbers and causes of death in thalassaemia major in the UK.

	**Period**						
**Cause of death**	1950 – 59	1960 – 69	1970 – 79	1980 – 89	1990 – 99	2000 – 03	Total

Iron overload		1	21	37	46	6	111
Anaemia	6	17	9	1			33
BMT complication				5	15	1	21
Infection		1	4	5	6	3	19
Malignancy		1	1		2	1	5
Other		2	2	8	4		16
Unknown					1		1

TOTAL	6	22	37	56	74	11	206

*% iron overload*	*0*	*4.5*	*56.8*	*66.1*	*62.2*	*54.5*	*53.9*

BMT = Bone marrow transplantation

### CMR and iron chelator

At the end of 2003, 40% of thalassemia patients born before 1980 had undergone T2* CMR. Of these patients, 36% were prescribed deferiprone, in the majority of cases following the results of the CMR scan showing cardiac siderosis. All remaining patients were taking deferoxamine only, as deferasirox was only introduced into clinical trials in the UK after 2003.

### Death rates

**Figure 1:**
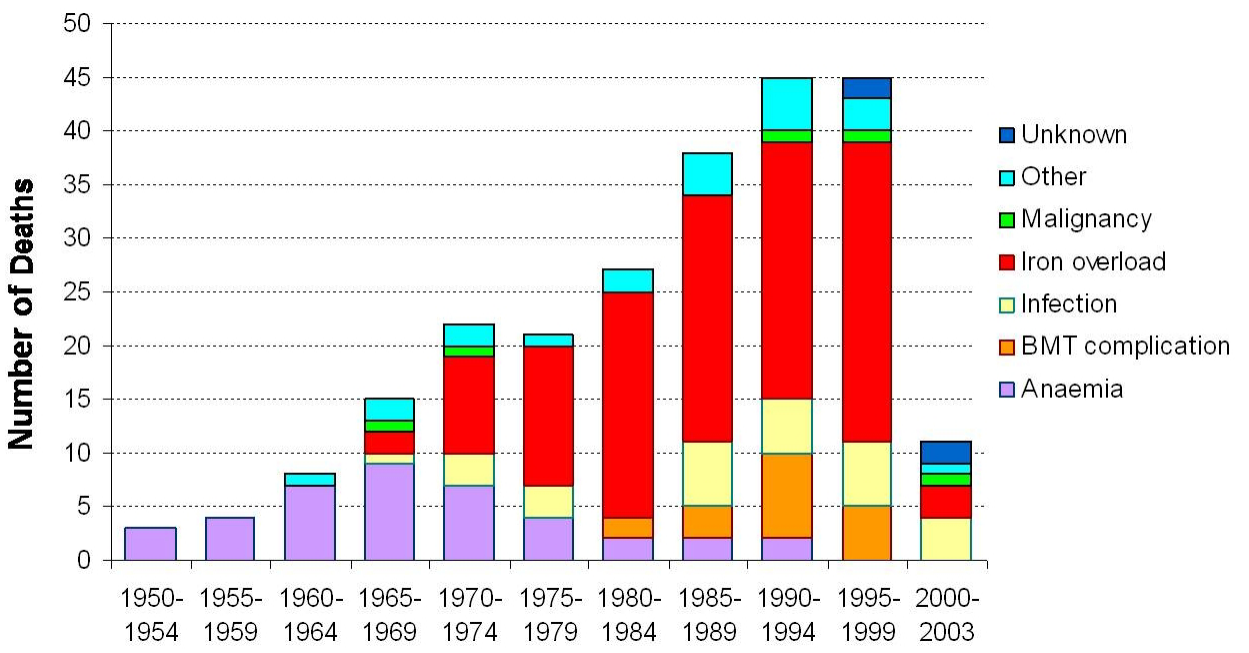
**Number of deaths of patients with thalassaemia major in the UK by intervals.** The number of deaths in the 2000–2003 interval represents deaths during 4 years, and in all the other groups the number of deaths is over 5 years. Iron overload replaced anaemia as the commonest cause of death after 1970, when adequate transfusion schemes became the norm. Iron chelation therapy by subcutaneous infusion of deferoxamine was standard practice after 1980. In 1999, T2* CMR was introduced in the UK, and doctors caring for thalassemia patients were informed of the high cardiac death rate and new options for iron chelation therapy. There has been a 71% reduction in the annualized death-rate from iron overload since 2000.

Tables 3, 4, 5 and 6 shows numbers of deaths, number of patient years, and death rates (deaths per 1,000 patient years) from all causes and from iron overload by decade. The final columns of Table 5 and 6 show a steady decrease in death rate from iron overload among patients 10–20 years old. However, in 1990–99 this was offset by an increased death rate among older patients, giving an average death rate from iron overload of 7.9 per 1000 patient years from 1980 to 1999. Since 2000 there has been a marked decrease in deaths from iron overload at any age, giving an average rate of 2.3 per 1,000 patient years (-71%, p < 0.05, figure 1). Table 7 compares average death rates by decade. There was no significant difference in death rates from all causes or from iron overload in the three decades starting in 1970, 1980 and 1990. Since the beginning of 2000 there has been a significant fall in death rate from all causes (-62%, p < 0.05).

**Table 3:** Death rates from all causes in thalassaemia major in the UK, by decade.

**All Causes**		Patient years							Deaths, all causes						
Year of birth	Births	Before 1960	1960–69	1970–79	1980–89	1990–99	2000–03	Total	Before 1960	1960 – 69	1970 – 79	1980 – 89	1990 – 99	2000 – 03	Total
Before 1960	55	263	454	312	186	140	48	1403	6	7	17	10	3	0	43
1960–69	166		842	1,452	1,205	948	346	4,793		15	15	32	19	2	83
1970–79	216			1,107	2,081	1,814	665	5,667			5	8	35	3	51
1980–89	212				1,234	1,982	750	3,966				6	15	5	26
1990–99	172					952	683	1,635					2	1	3
2000–2003	29						92	92						0	0

TOTAL	850	263	1,296	2,871	4,706	5,836	2,584	17,556	6	22	37	56	74	11	206

**Table 4:** Death rates from all causes per 1,000 patient years in thalassaemia major in the UK, by decade.

**All Causes**		Deaths, all causes per 1,000 patient years						
Year of birth	Births	Before 1960	1960 – 69	1970 – 79	1980 – 89	1990 – 99	2000 – 03	Total
Before 1960	55	22.8	15.4	54.5	53.8	21.4	0.0	30.6
1960–69	166		17.8	10.3	26.6	20.0	5.8	17.3
1970–79	216			4.5	3.8	19.3	4.5	9.0
1980–89	212				4.9	7.6	6.7	6.6
1990–99	172					2.1	1.5	1.8
2000–2003	29						0.0	0.0

TOTAL	850	22.8	17.0	12.9	11.9	12.7	4.3	11.7

**Table 5:** Death rates from iron overload per 1,000 patient years in thalassaemia major in the UK, by decade.

**Iron overload**		Patient years							Deaths from iron overload						
Year of birth	Births	Before 1960	1960–69	1970–79	1980–89	1990–99	2000–03	Total	Before 1960	1960 – 69	1970 – 79	1980 – 89	1990 – 99	2000 – 03	Total
Before 1960	55	263	454	312	186	140	48	1403	0	1	14	6	2	0	23
1960–69	166		842	1,452	1,205	948	346	4,793		0	7	27	15	1	50
1970–79	216			1,107	2,081	1,814	665	5,667			0	4	23	1	28
1980–89	212				1,234	1,982	750	3,966				0	6	4	10
1990–99	172					952	683	1,635					0	0	0
2000–2003	29						92	92						0	0

TOTAL	850	263	1,296	2,871	4,706	5,836	2,584	17,556		1	21	37	46	6	111

**Table 6:** Death rates from iron overload in thalassaemia major in the UK, by decade.

**Iron overload**		Deaths from iron overload per 1,000 patient years						
Year of birth	Births	Before 1960	1960 – 69	1970 – 79	1980 – 89	1990 – 99	2000 – 03	Total
Before 1960	55	0.0	2.2	44.9	32.3	14.3	0.0	16.4
1960–69	166		0.0	4.8	22.4	15.8	2.9	10.4
1970–79	216			0.0	1.9	12.7	1.5	4.9
1980–89	212				0.0	3.0	5.3	2.5
1990–99	172					0.0	0.0	0.0
2000–2003	29						0.0	0.0

TOTAL	850	0.0	0.8	7.3	7.9	7.9	2.3	6.3

**Table 7:** Change in death rates in thalassaemia major in the UK (* p < 0.05 for 2000–03 compared with 1990–99 or 1980–99).

					Deaths per 1,000 patient years *(95% confidence Interval)*		
Decade	All deaths	Iron overload deaths	Deaths other causes	Patient years	All deaths	Iron overload deaths	Deaths other causes

Before 1960	6	0	6	263	22.8 *(8.4,49.7)*	0.0 *(0.0,14.0)*	22.8 *(8.4,49.7*)
1960–69	22	1	21	1,296	17.0 *(10.6,25.7)*	0.8 *(0.0, 4.3)*	16.2 *(10.0,24.8)*
1970–79	37	21	16	2,871	12.9 *(9.1,17.7)*	7.3 *(4.5,11.1)*	5.6 *(3.2,9.1)*
1980–89	56	37	19	4,706	11.9 *(9.0,15.4)*	7.9 *(5.5,10.8)*	4.0 *(2.4,6.3)*
1990–99	74	46	28	5,836	12.7 *(10.0,15.9)*	7.9 *(5.8,10.5)*	4.8 *(3.2,6.9)*
2000–2003	11	6	5	2,584	4.3 *(2.1,7.6)*	2.3 (0.9,5.1)*	1.9 *(0.6,4.5)*

TOTAL	206	111	95	17,556	11.7 *(10.2,13.5)*	6.3 *(5.2,7.6)*	5.4 *(4.4,6.6)*

1980–99	130	83	47	10,542	12.3 *(10.3,14.6)*	7.9 *(6.3,9.8)*	4.5 *(3.3,5.9)*
2000–03	11	6	5	2,584	4.3 *(2.1,7.6)*	2.3 (0.9,5.1)*	1.9 *(0.6,4.5)*

## Discussion

### Reduced Mortality in Thalassemia

The data show a significant improvement in survival in thalassaemia major since 1999. In the younger patient group this is largely due to improved survival following bone marrow transplantation, and in older patients (born before 1980) it is dominated by decreased mortality from cardiac iron overload. It is likely that multiple factors have contributed to the improving survival of older patients. Whilst their relative contributions cannot be determined with certainty, the most relevant factor appears to be the introduction of the use of T2* CMR for assessing cardiac iron loading, thereby identifying patients at highest risk who require intensified iron chelation treatment, this being linked to improved chelation and the increasing use of the oral iron chelator deferiprone [18,19]. Other contributing factors include the improved application of conventional methods for assessing cardiac function and intensive deferoxamine therapy [20,21], communication of the need for vigilance of heart disease to treating doctors, the referral of patients to expert centres for assessment, and the promotion of new developments to patients, nurses and doctors by the UK Thalassaemia Society [22].

A similar change with improved survival has been reported from Italy, where T2* CMR has been available since 2002, and has been definitely linked to deferiprone use [23,24]. A comparable improvement in survival has also been observed in Cyprus, where T2* CMR was introduced in 2004, and where the most significant change in treatment has also been the increasing use of deferiprone, usually in combination with deferoxamine [25]. In both these countries, the use of T2* CMR may also have contributed to improved identification and treatment of patients with myocardial siderosis. These observations are consistent with the greater tolerability of an oral drug, the superior ability of deferiprone compared with deferoxamine to remove iron rapidly from the heart [17], and the substantial effect of deferoxamine and deferiprone when used in combination for increasing iron excretion [26,27]., The use of T2* CMR has also highlighted the large number of patients with cardiac iron overload who are on chronic deferoxamine treatment [12,19], who are at risk particularly when myocardial T2* is less than 10 ms [28], sometimes even with good compliance [13]. The widespread application of T2* CMR has been realised [29,30], and when combined with appropriate chelation management [18,28,31,32], the current observations optimistically suggest that, with sustained collaborative effort, it may now be possible to eliminate death from transfusional iron overload.

### Changing Life Expectancy

**Figure 2:**
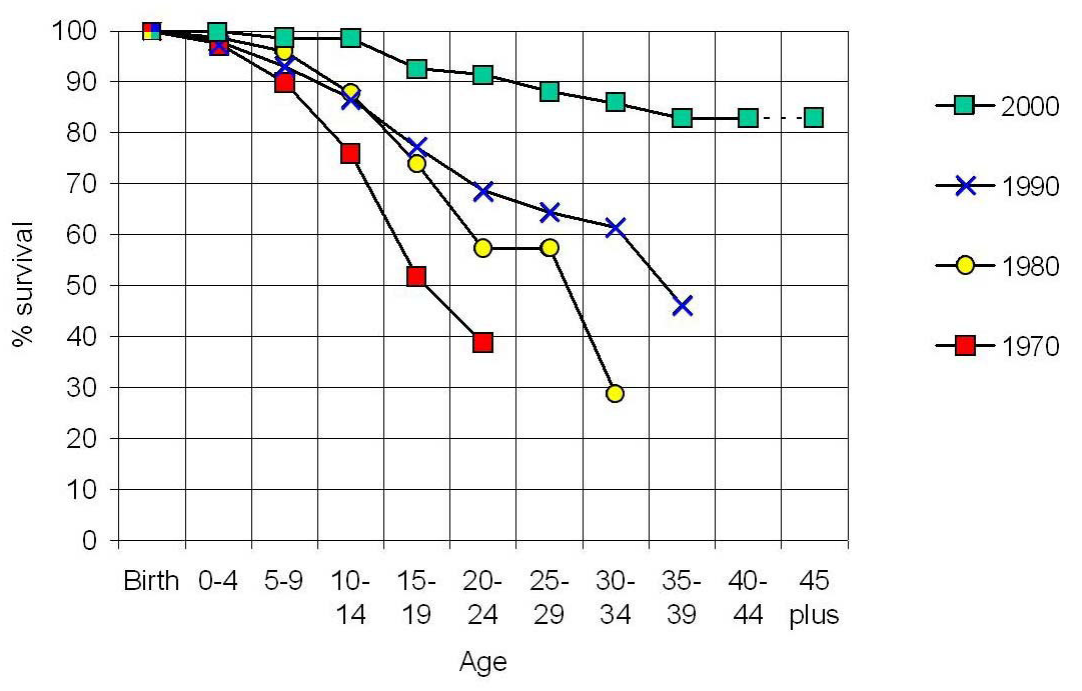
**Comparison of life expectancy for patients who were alive at the beginning of 1970, 1980, 1990 and 2000, based on mortality in the subsequent five years (four years for the period 2000–2003).** Curves are constructed by calculating the proportion of patients in each group who were alive at the time indicated and were still alive at the end of the next 5 years. For the four year interval 2000–2003, mortality was multiplied by 1.25 to adjust to 5 years. The calculation shows an average life-expectancy of 17 years in 1970, 27 years in 1980 and 37 years in 1990. Since 2000 over 80% of patients have a life expectancy of more than 40 years. It is still not possible to estimate ultimate life-expectancy, and the prognosis for older patients remains "open-ended".

Survival data is often presented as Kaplan Meier curves, which show cumulative mortality in patient cohorts born in specified periods [7,20,33], but this focus on attrition rate can be pessimistic, especially in clinical discussions with older patients. In practice, the more optimistic picture of the current life-expectancy of living patients, as calculated by life insurance companies, is more useful for patients and health workers. Figure 2 compares life expectancy for patients who were alive at the beginning of 1970, 1980, 1990 and 2000, based on mortality in the subsequent five years (four years for the period 2000–2003). The calculation shows an average life-expectancy of 17 years in 1970, 27 years in 1980 and 37 years in 1990. Since 2000, over 80% of patients have a life expectancy of more than 40 years. It is still not possible to estimate ultimate life-expectancy, and the prognosis for older patients remains "open-ended".

### Non-Cardiac Causes of Death

No deaths have occurred in the UK as a direct result of the use of either deferoxamine or deferiprone. However, several deaths indirectly related to deferoxamine therapy have been reported – two due to infection with Yersinia enterocolitica (which uses the iron chelate ferrioxamine as an iron source) [34], and one from cardiac tamponade due to a misplaced Hickman line. More time would be needed to assess the likelihood of death indirectly related to deferiprone.

With a reduction in deaths from iron overload, infection may become a leading cause of death in thalassaemia in the future. Splenectomy increases risk of infection with Pneumococcus and Haemophilus influenzae and deferoxamine therapy increases risk of infection with Yersinia enterocolitica, and there have been at least 3 deaths from these infections. However, the most frequently isolated organism was Klebsiella. An increased risk of Klebsiella infection in thalassaemia has previously been reported from South East Asia [35,36], and some forms of Klebsiella can use deferoxamine as an iron source [37], but it remains to be clarified whether Klebsiella infection is related to iron chelation therapy. There are two main risks for thalassaemics with a Klebsiella infection: acutely ill patients are typically admitted to the nearest hospital where there is usually little experience with thalassaemia, and current treatment practice focuses on a likely gram positive infection. Patients and doctors should be alert to the fact that an acutely ill patient with thalassaemia requires antibiotics active against both gram negative and gram positive organisms. Hepatocarcinoma is also a growing problem for hepatitis C positive patients, and improved antiviral treatments are needed. Fortunately, transmission of hepatitis C by blood transfusion is now very rare, so this risk may be limited to older patients.

## Conclusion

The control of cardiac iron overload as the major cause of death in thalassaemia major has been greatly improved by the deployment of T2* CMR, which makes cardiac siderosis visible both to clinicians and patients, in combination with appropriate alterations in iron chelator treatment. This includes the wider deployment of the oral iron chelator deferiprone both as monotherapy and in combination with deferoxamine. These two factors are likely to be the main drivers for the improvement in mortality identified in the UK thalassemia register. Should cardiac disease be conquered, then attention will inevitably need to be focussed on other causes of mortality, notably infections. These data suggest that widespread use of T2* CMR [38], or newer related sequences [39-41], has great potential to reduce the cardiac mortality in chronically transfused beta-thalassemia major.

## Authors' contributions

BM, MK, MD and DI participated in conception, design, and obtaining of funding for the registry, and drafted the manuscript with participation in analysis and interpretation of data. MAW and DJP participated in drafting the manuscript, analysing the CMR data, and interpreting the data. All authors read and approved the final manuscript.
